# A systematic review of left mainstem quadfurcation anatomy ‘The Starfish Sign’: an incidental finding or a variant of concern?

**DOI:** 10.1093/ehjimp/qyag157

**Published:** 2026-09-03

**Authors:** Anas Musa, Zoubia Fathima, Azza Mohamedelamin, Usama Al Bastaki, Edouard Long, Ronak Rajani

**Affiliations:** Guy’s and St Thomas’ NHS Foundation Trust, London SE1 7EH, UK; Cardiology Department, Rashid Hospital, Dubai Health, 315 Umm Hurair Second, Street 2, Dubai, UAE; Cardiology Department, Rashid Hospital, Dubai Health, 315 Umm Hurair Second, Street 2, Dubai, UAE; Cardiology Department, Rashid Hospital, Dubai Health, 315 Umm Hurair Second, Street 2, Dubai, UAE; Radiology Department, Rashid Hospital, Dubai Health, 315 Umm Hurair Second, Street 2, Dubai, UAE; Guy’s and St Thomas’ NHS Foundation Trust, London SE1 7EH, UK; School of Biomedical Engineering and Imaging Sciences, King’s College London, London SE1 7EH, UK; Guy’s and St Thomas’ NHS Foundation Trust, London SE1 7EH, UK; School of Biomedical Engineering and Imaging Sciences, King’s College London, London SE1 7EH, UK

**Keywords:** coronary computed tomography, coronary anomaly, left mainstem, quadrifurcation anatomy, systematic review

## Abstract

Left mainstem coronary artery quadrifurcation (LMQ) is a coronary variant with a wide-ranging estimated prevalence of 1.8–9.6%. Although often considered an incidental and benign finding, there is a dearth of literature to support this notion owing to suboptimal clinical characterization of this phenomenon. The aim of the current manuscript was to perform a systematic review of LMQ and to describe its prevalence, associated demographics and reported clinical outcomes. We furthermore propose new nomenclature for this finding. A systematic review was conducted in accordance with the PRISMA 2020 guidelines. Eligible studies were detected for inclusion into the review using web-based sources (i.e. PubMed, Google Scholar) over a 20-year period from January 2005 to September 2025. Search terms included ‘left mainstem quadrification/quadrifurcation/quadfurcation/tertrafurcation, coronary artery branches, coronary artery anomalies, and coronary intervention’ using left main quadrifurcation and its wording variants. Where available, cardiovascular risk factors and clinical outcomes were recorded. Statistics were performed using SPSS V24 and R V3.4. LMQ prevalence was estimated by pooled analysis of cadaveric series using a DerSimonian–Laird random-effects model. *I*^2^, Tau, and *Q* statistics were also calculated to assess heterogeneity. Publication bias was assessed by Funnel plot and Egger’s regression. In an exploratory analysis, comparative statistics were performed to assess differences between coronary angiography (CAG) and coronary computed tomographic angiography (CCTA) detection using the χ^2^ test, Student’s *t-*test, and Fisher’s exact test, as appropriate. A *P-*value < 0.05 was taken to be significant by convention. Nineteen studies were identified, from which two were excluded. The total number of patients evaluated was 757. There were eight cadaveric series (total of 747 cases of which 44 had LMQ quadrification) and the pooled prevalence was 4.5% (95% CI: 2.53−6.47%). There was moderate heterogeneity (*I* = 43.2%, *Q* = 12.33, *P* = 0.090, *T* = 0.00034) with Egger’s regression indicating asymmetry (intercept = −2.12, *P* = 0.001), suggesting small-study effects. Although limited by study numbers (*k* = 8), the overall risk of bias was low to moderate with consistently low bias in sample selection and measurement reliability and moderate risk in reporting completeness. There was a trend to earlier detection by CCTA compared with CAG (55.3 ± 10.8 years vs. 70 ± 8.5 years, *P* = 0.056; 95% CI: 0.5–31.5). Clinical characterization of LMQ anatomy was inconsistent as was the documentation of clinical outcomes. In conclusion, Pooled analysis of post-mortem studies indicates the prevalence of LMQ anatomy to be 4.5%. Despite several prior reports, clinical characterization of this phenomenon remains poor. We propose a novel name for this phenomenon ‘LMCA Starfish variant’ along with standardized coronary branch nomenclature to help identify and increase awareness of this variant.

## Graphical Abstract

**Graphical Abstract:**
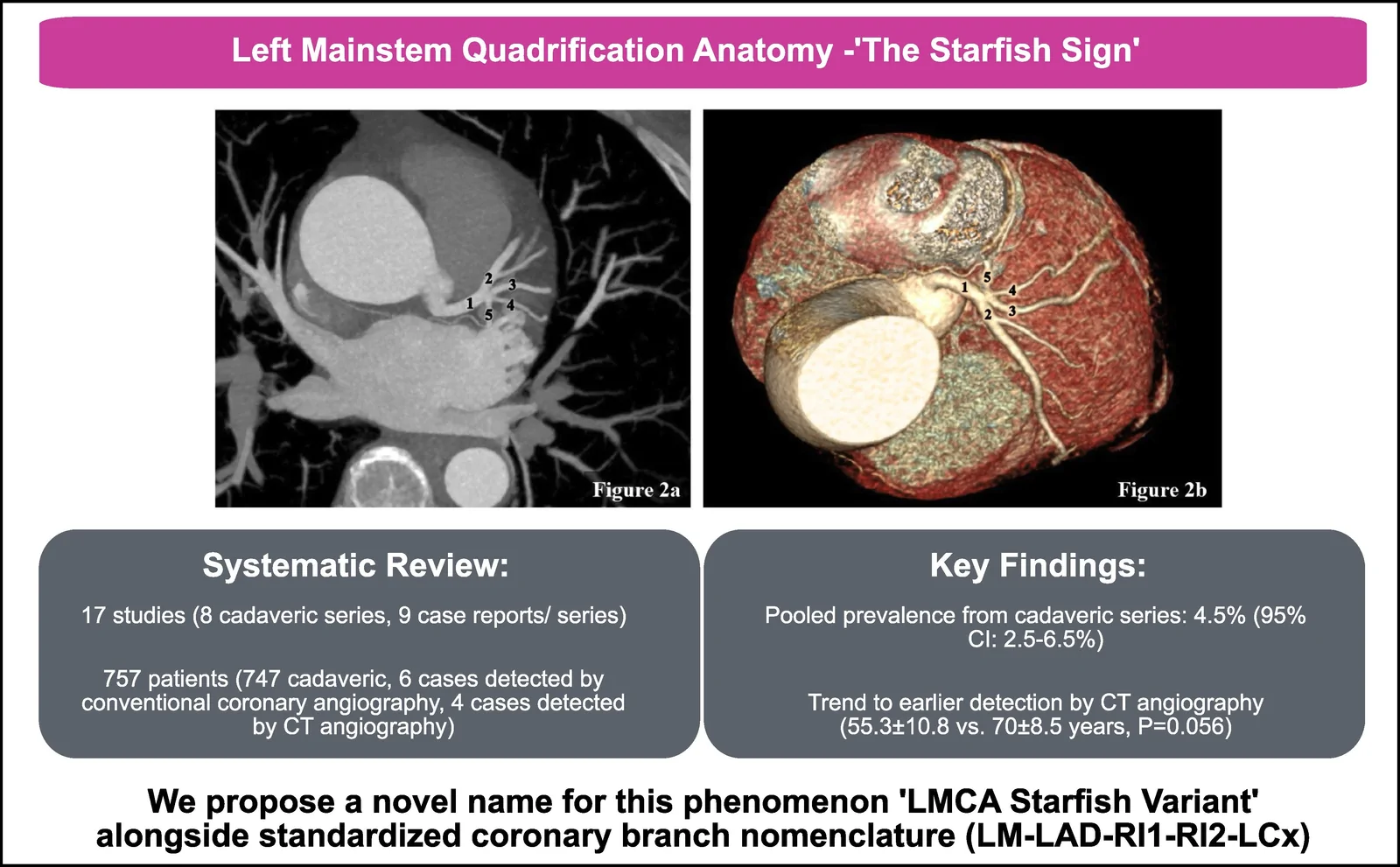

## Introduction

The left mainstem artery describes the common vessel that arises from the left coronary cusp to supply the left coronary circulation. This bifurcates into the left anterior descending (LAD) artery and left circumflex (LCX) artery in 70–80% of cases.^1^ Another frequent configuration is that of trifurcation anatomy, which occurs in 6.7% to 52.2% of cases. In this instance, an additional vessel known as the ramus or intermediate artery passes between the LAD and LCX arteries.^2^ In a small percentage of cases, an additional vessel to the ramus branch can arise from the distal margin of the left mainstem giving rise to a phenomenon known as left mainstem quadrifurcation (LMQ) anatomy. This finding is often confused with anatomical descriptions of ‘an early or high arising first diagonal branch’ or a ‘high arising first obtuse marginal branch’.

Despite an increase in the use of coronary imaging, the true prevalence of LMQ anatomy remains uncertain, alongside whether this is an incidental/benign entity or may have significant clinical consequences. The principal aim of the current manuscript was therefore to conduct a systematic analysis of LMQ anatomy to understand its true prevalence, demographic characteristics, and clinical course.

## Methods

An extensive literature search was conducted on PubMed/PMC, Embase, Google Scholar, and other medical websites such as Radiopedia, PCR-online, and HMPGlobal using the identical terms ‘left main quadrification/quadrifurcation/quadfurcation/tertrafurcation, coronary artery branches, coronary artery anomalies, and coronary intervention’. We included case reports and case series published over a 20-year period from 2005 to 2025 that described LMQ and coronary intervention in these cases (if any). Retrieved data were filtered for duplications and assessed for quality and bias by two independent researchers. Reports of cases with bifurcation, trifurcation and pentafurcation of the LMCA, alongside those with missing data, were excluded. A flow chart was produced in line with PRISMA 2020 guidelines (*Figure 1*).

**Figure 1:**
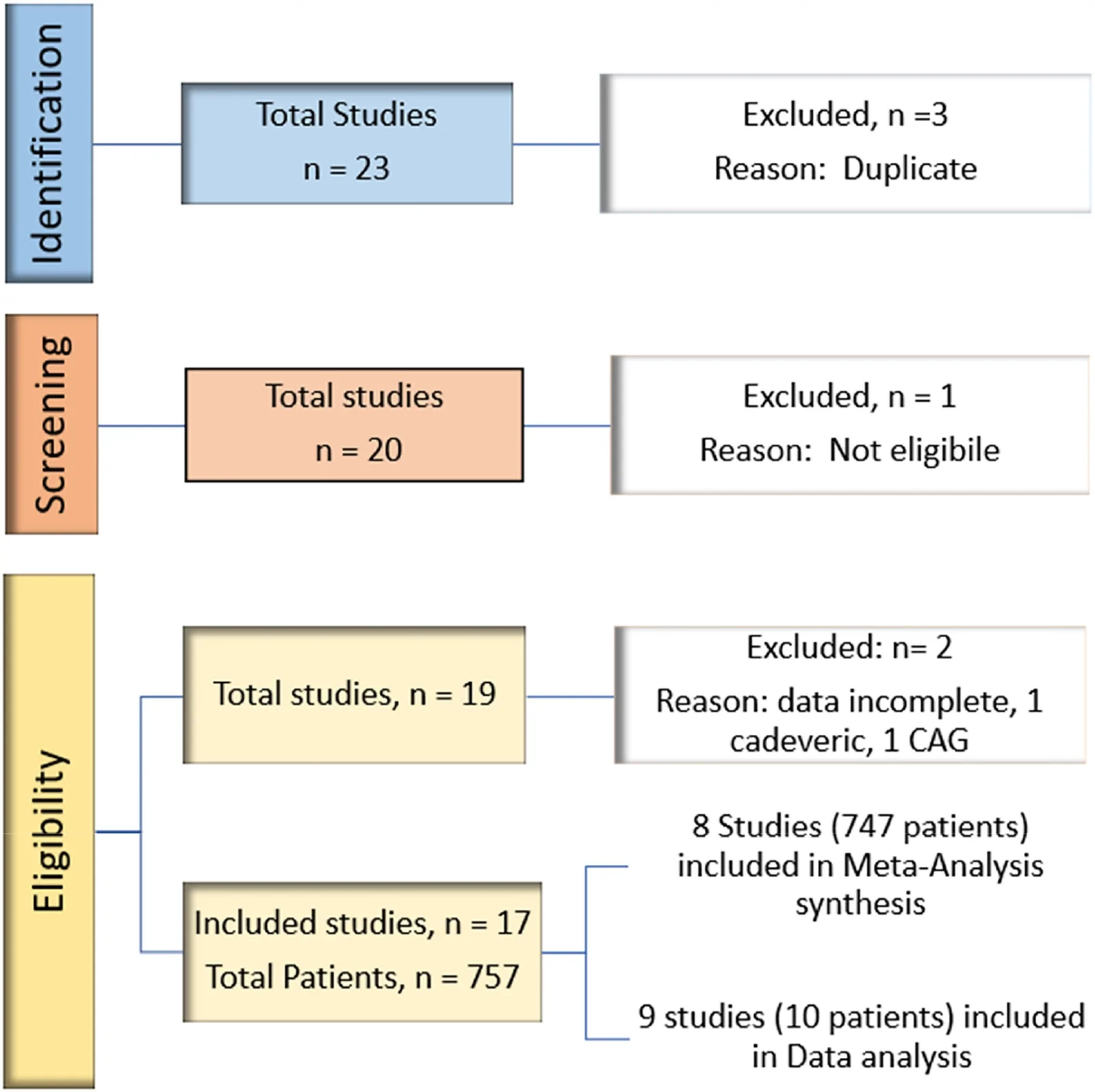
Prisma flowchart describing the study inclusion and exclusion criteria.

A complete retrieval of full texts and abstracts was collated and the key findings, demographics, co-morbid conditions, authors, year of publication, country, study samples, detection mode, sample size, and coronary artery findings were recorded onto a database for analysis. We reached out to one author who supported us with their case full text (see Acknowledgement). The cases were then grouped into three major groups according to their mode of detection: (i) post-mortem/cadaveric source; (ii) conventional coronary angiography (CAG), and (iii) coronary computed tomographic angiography (CCTA).

We thereafter pooled and analysed cases/case series detected by either CAG or CCTA to gain more insight into relevant associated factors.

### Statistical analysis

The prevalence of LMQ was calculated based on the prevalence reported in post-mortem case series; cases identified by CAG or CCTA were not included in this calculation. We calculated study-level proportions and exact (Clopper–Pearson) 95% confidence intervals, and a DerSimonian–Laird random-effects model was used to pool prevalence. Heterogeneity was quantified by *I*^2^ and the Cochran *Q* statistic. Thresholds of 25%, 50%, and 75% were used to represent low, moderate, and high study heterogeneity, respectively. Publication bias was assessed using funnel plot inspection and Egger’s regression on logit-transformed proportions.

Most of the cases detected by CAG or CCTA were documented on case reports, and these were separately analysed for demographic data, risk factors, lesion characteristics, and management, stratified by the mode of detection. In an exploratory descriptive analysis, information was grouped to calculate the percentage of risk factors along with age and gender distribution. Continuous variables were summarized and compared by Student’s *t*-test or Mann–Whiteny test, as appropriate, and summarized as mean ± SD, while χ^2^ or the exact Fisher’s test was used to infer and summarize categorical variables.

The risk of bias and quality of results methodology for the included post-mortem and cadaveric studies were evaluated using a modified anatomical quality assessment checklist adapted from the Newcastle–Ottawa Scale and the Anatomical Quality assessment tool (AQUA). Each study was assessed independently with regards to methodology and sample selection, reliability of measured variables, and completeness of reporting; and consensus resolution in case of disagreement between assessors was decided. The evidence was graded from low to high risk of bias and overall risk of bias of each study was determined by the dominating level of risk and the collective risk distribution bias was calculated for Tau and *I*^2^ statistic to determine meta-analytic heterogeneity. Results were interpreted at level of significance (*P*) of less than 0.05 and all analysis was performed using the SPSS system version 24 and R Version 4.3.

## Results

Our search resulted in a total of 17 eligible case series and reports amounting to a total of 757 evaluated patients. There were eight post-mortem case series (*Table 1*), and nine case reports identified by CCTA or CAG as having LMQ anatomy (*Table 2*). The risk of bias of individual studies included for cadaveric/post-mortem analysis for calculation of the prevalence is summarized in *Table 3*.

**Table 1:** Cases of left main quadrification detected by anatomical dissection

Ref.	Total number of cases dissected	Actual number	Percentage with LMQ (%)
**^3^**	154	9	5.8
**^4^**	64	5	7.8
**^5^**	208	20	9.6
**^6^**	55	1	1.82
**^7^**	50	1	2
**^8^**	1	NA	NA
**^9^**	76	3	4
**^10^**	40	1	2.5
**^11^**	100	4	4
**Total**	**748**	**44**	**6**

Ref., reference number.

**Table 2:** Identification of LMQ artery during CAG or CTCA and management of lesions

Ref.	Age	Gender (M/F)	CV risk factors	Symptoms	Test	Lesions		Tx
**^12^**	63	F	HTN Dysl. Smoking	CP Dyspnoea + Isch. Test	CAG	Non-obstructive CAD LAD and RCA		None
**^13^**	65	M	NA	ACS	CAG	**Case 1**	Distal LMCA Ostial (LCx) artery 2×RI	CABG
	82	M	IHD	ACS	CAG	**Case 2**	Ostial LCX	PCI
**^14^**	83	M	DM HTN CCF VaD. IHD	CP	CAG	Multi vessel disease, including the point of quadrifurcation		PCI
**^15^**	69	M	NA	ACS	CAG	LMCA LAD LCx 2 × RI		PCI
**^16^**	63	M	HTN Dysl. BMI	CP + Isch. Test	CAG	LMCA aneurysm with quadrifurcation No significant stenosis		None
**^17^**	70	M	HTN	CP ACS Cardiogenic shock	CAG	LMCA		PCI
**^18^**	45	M	NA	CP Dyspnoea	CTCA	No significant stenosis		NA
**^19^**	70	F	NA	ACP Dyspnoea	CTCA	No significant stenosis		NA
**^20^**	50	F	NA	CP	CTCA	No significant stenosis		NA
**NA**	56	F	IGT BMI Dysl.	CP	CTCA	No significant stenosis		NA

Ref., reference number; M, male; F, female; HTN, hypertension; Dysl., dyslipidaemia; CCF, congestive cardiac failure; VaD, vascular disease; IGT, impaired glucose tolerance; IHD, ischaemic heart disease; BMI, elevated body mass index; + Isch. Test, positive functional test for ischaemia; ACP, acute chest pain; CP, chest pain; CAG, invasive coronary angiography; CCTA, coronary computed tomographic angiography; LMCA, left mainstem; LAD, left anterior descending artery; LCx, left circumflex artery; RI, ramus intermedius artery; RCA, right coronary artery; PCI, percutaneous coronary intervention; CABG, coronary artery bypass grafting; NA, not applicable (case from our institution).

**Table 3:** Modified risk-of-bias assessment for included cadaveric studies, risk is calculated at 25%, 50%, and 75% and graded as low, moderate, and high risk, respectively

*Study*	*Sample selection*	*Measurement reliability*	*Reporting completeness*	*Overall*
*Ballesteros 2008^3^*	Low	Low	Moderate	Low-Moderate
*Dombe 2011^4^*	Low	Low	Moderate	Low-Moderate
*Ogeng’o 2014^5^*	Low	Low	Moderate	Low-Moderate
*Lakshmiprabha 2018^6^*	Low	Low	Moderate	Low-Moderate
*Apsara 2019^7^*	Low	Low	Moderate	Low-Moderate
*Jaishree 2021^9^*	Low	Low	Moderate	Low-Moderate
*Morais 2024^10^*	Low	Low	Moderate	Low-Moderate
*Agnihotri 2024^11^*	Low	Low	Moderate	Low-Moderate

The overall pooled prevalence from the post-mortem case series of LMQ was 4.5% (95% CI: 2.56–6.47). There was moderate interstudy heterogeneity (*I*^2^ = 43.2%, *Q* = 12.33, *P* = 0.090), whilst Egger’s test on logit-transformed proportions indicated asymmetry (intercept = −2.119, *P* = 0.001). Considering the small number of studies this should be interpreted with caution (*Table 4*).

**Table 4:** Meta-analysis statistics and calculation of prevalence and confidence interval of LMQ from post-mortem sources

*Test parameter*	*Value*
*K (studies)*	**8**
*Total sample size*	**747**
*Total cases*	**44**
*Pooled prevalence (random effects)*	**0**.**045**
*95% CI: lower*	**0**.**025**
*95% CL: upper*	**0**.**065**
*Q statistics*	**12**.**326**
*Q P-value*	**0**.**090**
*I-squared (%)*	**43**.**208**
*Tau*	**0**.**000**
*Egger intercept*	**−2**.**119**
*Egger P-value*	**0**.**001**

In the exploratory descriptive analysis, nine case reports/series that fulfilled our search criteria describing cases of LMQ detected by CCTA or CAG with appropriate clinical characterization results are presented in *Table 5*. The baseline characteristics of the two groups revealed that the cases detected by CCTA were significantly younger with a female predominance. Cases of LMQ detected by CCTA were far younger than those detected by CAG with an average difference of more than 15 years (*P* = 0.056). Despite this not reaching statistical significance, the effect size suggests that LMQ is frequently detected at a younger age by CCTA.

**Table 5:** Comparison of cases of left main quadrifurcation detected by CAG and CTCA: presenting proportions and number

Parameter	Group 1: cases detected by CAG (*N*)	Group 2: cases detected by CTCA (*N*)	*P-*value
**Total number of cases**	7 (63.6%)	4 (36.4%)	**—**
***Baseline characteristics***			
** Mean age ± SD (in years)**	70.71 ± 8.5	55.25 ± 10.8	0.056
** Age difference (95% CI)**	Mean: 15.5, 95% CI: 0.5–31.5 years		—
** Male sex**	85.7% (6)	25% (1)	0.088
***Previous comorbidities***			
** Hypertension**	57.14% (4)	0% (0)	0.194
** Diabetes mellitus**	14.30% (1)	0% (0)	1.000
** Ischaemic heart disease**	42.90% (3)	0% (0)	0.236
** Dyslipidaemia**	28.60% (2)	25% (1)	1.000
** History of smoking**	14.30% (1)	0% (0)	1.000
** Obesity**	14.30% (1)	25% (1)	1.000
** Heart failure**	28.60% (2)	0% (0)	0.491
***Presenting complaint of the patient at the time of coronary assessment***			
** Chest Pain**	100.00% (7)	100% (4)	—
** Shortness of breath**	28.60% (2)	75% (3)	0.14
***Other investigations done prior to coronary assessment***			
** ECG**	71.43% (5)	50% (2)	0.576
** Troponin**	28.60% (2)	0% (0)	0.491
** Nuclear imaging**	28.60% (2)	0% (0)	0.491
***Coronary assessment***			
** Presence of coronary lesions**	85.71% (6)	0% (7)	0.015
** LMCA involvement**	57.14% (4)	0% (0)	0.194
** Involvement of 3–4 branches**	57.14% (4)	0% (0)	0.023
** Involvement of 1–2 branches**	28.60% (2)	0% (0)	
** Involvement of 0 branches**	14.30% (1)	100% (4)	

Patients who had LMQ detected by CAG had a higher proportion of traditional cardiovascular risk factors; however, there was no statistical difference between the two groups. All patients presented with symptoms of chest pain in both groups. As most of the patients detected by CAG presented with acute coronary syndrome, this group of patients frequently underwent more investigations to establish the diagnosis prior to coronary assessment. Upon evaluation of the coronary arteries, none of the cases detected by CCTA had significant coronary lesions. In contrast, cases detected by CAG had a significantly higher presence of lesions in the coronaries, with most of the cases having lesions in LMCA and three or more branches of the quadrifurcation.

## Discussion

There are several important observations from the current study. First, despite an increasing use of coronary imaging, there remains few systematic reports that detail the prevalence, nomenclature, or clinical significance of LMQ anatomy. Secondly, from the existing limited literature, our data suggest an overall prevalence of 4.5% of LMQ anatomy in selected cohorts of patients. Thirdly, we noted LMQ is often detected at an earlier age by CCTA and later by CAG. Finally, in those patients presenting with symptoms at CAG, disease within the left mainstem or quadrification branches was common.

This systemic analysis showed a satisfactory methodological quality of analysed cadaveric reports, with predominance of low-to-moderate risk across evaluated studies. This supports the reliability of the reported pooled prevalence of 4.5% (CI: 2.56–6.47%), despite inherent variability between anatomical reports. We observed a moderate heterogeneity on anatomical data series (*I*^2^ = 43.2%, *Q* = 12.33, *P* = 0.090, *T* = 0.00034), which suggests inherent study differences with regard to methodology and population characteristics may contribute to the observed variances rather than random error alone. This is supported by the observation that studies with rigorous dissection protocols and clear reporting methodology tended to cluster around 4.5%, whilst smaller and older studies lay on the higher or lower prevalence sides; a pattern that may also explain the minor inconsistency in sampling and measurements and indeed reflect a real anatomical diversity of reported cases, rather than systemic analytic bias (*Figure 2*).

**Figure 2:**
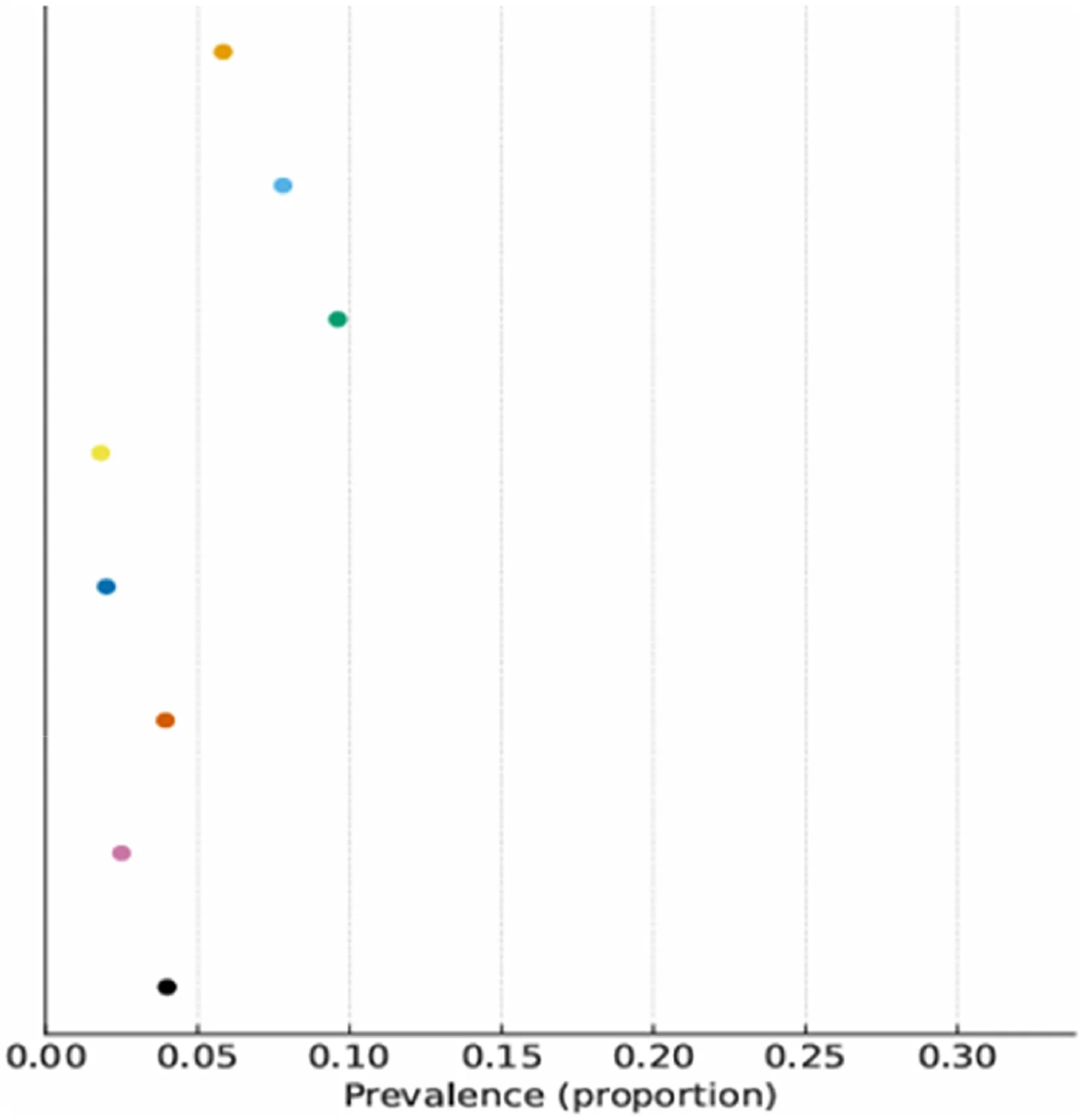
Forest logit-plot of LMQ prevalence around a calculated prevalence of 4.5% (95% CI 2.56–6.47%).

Upon review of prior published case reports and series of LMQ anatomy, we observed a tendency for LMQ anatomy to be detected in younger individuals by CCTA compared to older individuals where this finding was confirmed by CAG. Not surprisingly, there was limited evidence of early atheroma in this younger cohort of patients. This finding was as anticipated and reflects the emergence of CCTA over the last 20 years as the first line investigation of choice to evaluate younger and lower risk patients with stable chest pain.^21,22^

Interestingly in those patients who underwent CAG, disease was commonly observed within the left mainstem and the early segments of the quadrification branches. Although it remains difficult to derive any firm conclusions from this finding, it remains physiologically plausible that LMQ anatomy may predispose to atherogenesis and may not be as benign entity as it is often considered to be. Indeed, the presence of the fourth branch increases the angulation between the LAD and LCX arteries, resulting in increased regions of low wall shear stress and oscillatory flow, which may promote flow disturbances that predispose to plaque formation and growth.^23–26^ Neverthtless, it should be remembered that patients referred for CAG are usually older and symptomatic and thus this finding may reflect a degree of selection bias.

An awareness of LMQ anatomy may also be of value to those patients requiring revascularization by coronary artery bypass grafting of percutaneous coronary intervention. Quadrification plaque lesions may be complex and may require extensive surgical planning with regards to the number of grafts, insertion points, and types of bypass grafts required prior to surgery. Similarly, percutaneous management of these lesions may be challenging, as procedural technique is influenced by the size, number, and the angulation of the respective coronary branches. The morphological characteristics and the location of the coronary lesion also significantly impact upon procedural decisions.^12–17^

The current systematic review was prompted by the author’s own detection of LMQ anatomy by CCTA during their clinical practice (*Figure 3*). It was noted that despite this phenomenon being observed relatively frequently, there was no universal nomenclature to describe LMQ anatomy or to convey this finding to referring clinicians. The labelling of branches was inconsistent across prior reports with additional branches being labelled as ‘dual rami intermedius’ and other reports replacing the second ramus branch with an ‘early arising diagonal’ or ‘obtuse marginal branch’ depending upon the proximity to the LAD and LCX arteries, respectively. During assessment of our case, we noted that the appearance of the LMCA quadrifurcation resembles the structure of a ‘Starfish’ due to the presence of the four branches of the quadrifurcation in addition to the left main coronary artery itself (*Figure 4*). Hence, we propose the term ‘Starfish LMCA variant’ to collectively describe this anatomy to referring clinicians. Furthermore, we suggest a consistent approach to labelling the arms of the ‘Starfish LMCA variant’ as follows: LM-LAD-RI1-RI2-LCx in respective order (LM = left main; LAD = left anterior descending; RI1: first ramus intermedius; RI2: second ramus intermedius; LCx: left circumflex) (*Figure 3*). By assigning a consistent approach to describing this variant, we anticipate greater recognition. It is hoped that this will result in increased detection, clinical characterization, and an understanding of this anatomical presentation.

**Figure 3:**
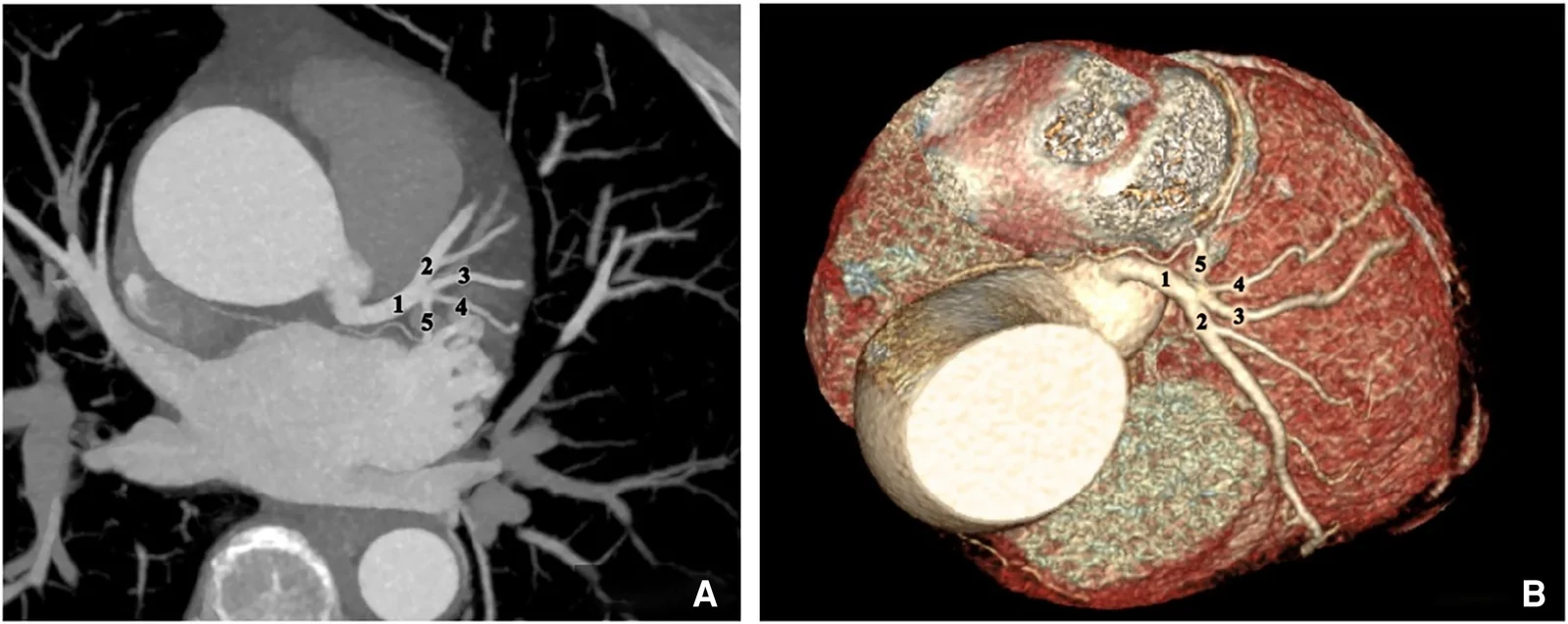
(*A*) Demonstrating quadrification of the LMCA with its branches. (*B*) Volume-rendered image showing quadrification of LMCA with its branches. Note the acute angle between LMCA and LCx and its retrograde path that may complicate coronary interventions. Also note the obtuse angle (>90 degrees) between LAD and LCx that is linked to reduced wall shear stress associated with increased plaque burden (1: left main coronary artery; 2: left anterior descending artery; 3: first Ramus intermedius branch; 4: second Ramus intermedius branch; 5: left circumflex coronary artery).

**Figure 4:**
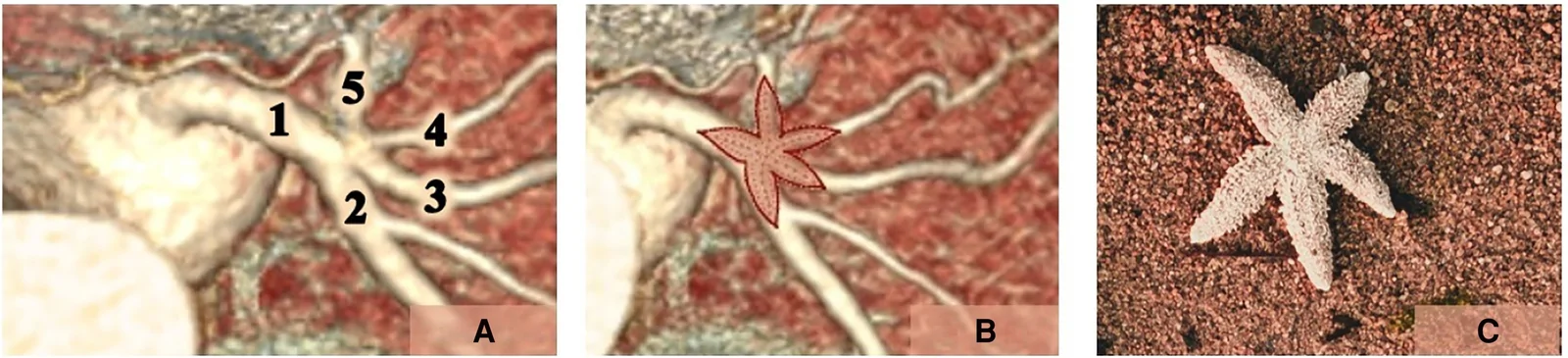
(*A*) Quadrifurcation of LMCA with its branches. (*B*) Starfish pattern superimposed over the volume rendered image of the LMCA Quadrifurcation. (*C*) Actual image of a starfish (reproduced with permission from Mr E. Y. Soysal via Pexels).

### Limitations

Techniques of detection of this coronary variant (‘Starfish LMCA’) have evolved over time. While most of the earlier cases reported in this systemic review (*Table 1*) were detected during post-mortem or cadaveric dissection; we noted that there was not enough information to form the basis for a registry owing to a lack of consistent patient-specific clinical data. Although we collated seven cases of ‘Starfish LMCA variant’ upon CAG, these cases presented with established coronary artery disease. Most of these cases (57%) demonstrated involvement of the left main along with at least three branches of the ‘Starfish LMCA’ with a complex coronary lesion. Given the small number of cases available for comparison between the CCTA and CAG groups, these comparisons are likely underpowered and should be interpreted as descriptive and hypothesis-generating. Although the authors were able to extract some clinical data from retrospective reports, the clinical information was incomplete and heterogenous. Moreover, patients referred for CAG are usually older and more symptomatic, which may introduce a degree of selection bias. Thus, the inference of pathological significance of LMQ anatomy is speculative and needs to be confirmed on larger prospective case series with longitudinal follow-up and more robust clinical characterization.

## Conclusion

In post-mortem studies, left mainstem quadrification anatomy was observed in 4.5% of patients. We propose a unique name for this variant (‘Starfish LMCA variant’) along with standardized coronary labelling nomenclature to increase awareness of this finding and inform referring clinicians. Robust registry data are warranted to further characterize the prevalence of this anatomical variant and whether it meaningfully impacts clinical outcomes.

## Data Availability

The data underlying this article will be shared on reasonable request to the corresponding author.

## Lead author biography

Anas Musa, MD, is a senior cardiologist specializing in advanced cardiovascular imaging, with a particular focus on cardiovascular magnetic resonance (CMR) and cardiac computed tomography (CCT). He holds the Arab Board in Cardiology, MRCP (UK), FRCP, and is a Fellow of the American College of Cardiology (FACC). His clinical and academic interests include multimodality cardiovascular imaging, coronary artery disease, cardiomyopathies, imaging in congenital and structural heart disease, and advanced applications of CMR and CCT. His research focuses on advancing cardiovascular imaging, coronary anatomy, and the integration of multimodality imaging into contemporary clinical practice. He is actively engaged in cardiovascular research, academic writing, and clinical education.
